# Diabetes and cancer: clinical implications for integrated metabolic–oncologic care

**DOI:** 10.3389/fonc.2026.1824109

**Published:** 2026-05-01

**Authors:** Vanessa Fuchs-Tarlovsky

**Affiliations:** 1 Unidad de Investigación en Nutrición Clínica, Dirección de Investigación, Hospital General de México Dr. Eduardo Liceaga, Mexico, Mexico; 2 Hospital Angeles Lomas, Huixquilucan de Degollado, Mexico; 3 Centro de Cancer, Clínica de Calidad de vida, Centro Médico ABC (The American British Cowdary Medican Center), Mexico, Mexico

**Keywords:** cancer risk, cancer therapy, diabetes mellitus, hyperglycemia, insulin resistance, metabolic oncology, metformin

## Abstract

Diabetes mellitus and cancer are among the most prevalent non-communicable diseases worldwide and increasingly coexist in clinical practice. Epidemiological evidence indicates that type 2 diabetes is associated with elevated risk of several malignancies, including hepatocellular, pancreatic, colorectal, endometrial, and breast cancers. At the same time, cancer and its treatments may significantly disrupt metabolic homeostasis and complicate glycemic control. Therapies such as glucocorticoids, PI3K inhibitors, and immune checkpoint inhibitors can induce hyperglycemia or precipitate new-onset diabetes. Metabolic dysregulation during cancer therapy has been associated with increased infection risk, treatment complications, and adverse clinical outcomes. This review summarizes the epidemiological evidence linking diabetes and cancer and discusses the clinical implications of their bidirectional interaction. The growing coexistence of both conditions highlights the need for integrated metabolic–oncologic care. Proactive glycemic monitoring, individualized antidiabetic therapy selection, and multidisciplinary management may improve treatment tolerance and long-term outcomes in patients with cancer and diabetes.

## Introduction

Diabetes mellitus and cancer are among the leading non-communicable diseases worldwide, imposing a substantial and growing global health burden. According to the International Diabetes Federation (IDF), approximately 589 million adults aged 20–79 years were living with diabetes in 2024, representing nearly one in nine adults globally. This number is projected to rise significantly in the coming decades, exceeding 850 million by 2050.

In parallel, cancer incidence continues to increase worldwide. Recent global estimates from the International Agency for Research on Cancer (IARC) indicate that nearly 20 million new cancer cases and 9.7 million cancer-related deaths occurred in 2022, with further increases anticipated due to population aging and epidemiologic transitions.

Beyond their independent prevalence, accumulating epidemiological evidence supports a clinically significant association between type 2 diabetes and the risk of several malignancies, including pancreatic, hepatocellular, colorectal, endometrial, and breast cancers The magnitude of this association varies by tumor type but appears biologically plausible given the presence of shared biological pathways, which are discussed in detail below.

Importantly, the relationship between diabetes and cancer is bidirectional. While diabetes may increase the risk of certain cancers and adversely affect oncologic outcomes, cancer and its treatments can also exacerbate metabolic dysfunction, induce hyperglycemia, and complicate glycemic management As cancer survival improves, a growing number of patients are living with both conditions concurrently, creating complex therapeutic and prognostic challenges that require coordinated care.

Despite expanding evidence describing this interaction, clinical guidance integrating metabolic and oncologic management remains limited. There is therefore a pressing need to translate epidemiological and mechanistic insights into practical recommendations that support integrated metabolic–oncologic care. As the prevalence of both diabetes and cancer continues to rise globally, clinicians increasingly encounter patients affected by both conditions simultaneously. Understanding the clinical implications of this interaction is essential to improve risk stratification, optimize metabolic management during cancer therapy, and ultimately improve patient outcomes.

This review synthesizes current evidence on the diabetes–cancer association and explores its clinical implications for cancer risk assessment, screening considerations, and therapeutic decision-making in patients with diabetes and malignancy.

### Epidemiologic magnitude of cancer risk in patients with type 2 diabetes

Multiple large-scale meta-analyses and cohort studies have demonstrated that type 2 diabetes is associated with an increased risk of several site-specific malignancies (1, 2). The magnitude of risk varies according to tumor type, with the strongest associations observed for hepatocellular carcinoma and pancreatic cancer (3–6). Relative risks for these malignancies range from 1.5 to 2.5, suggesting clinically meaningful effect sizes that warrant heightened vigilance in high-risk populations.

Moderate associations have been observed for colorectal, endometrial, breast, and bladder cancers, with relative risks generally ranging between 1.1 and 1.8 (7–10). While these effect sizes are smaller, their population-level impact is considerable given the high prevalence of diabetes worldwide, as summarized in **Table 1**.

**Table 1:** Site-specific cancer risk associated with type 2 diabetes based on pooled estimates from observational studies and meta-analyses.

Cancer type	Relative risk	Strength	Key references
Pancreatic	1.5–2.0	Strong	(11, 12)
Hepatocellular	2.0–2.5	Strong	(11, 13)
Colorectal	1.2–1.3	Moderate	(7, 11)
Endometrial	1.6–1.8	Strong	(8, 11)
Breast	1.1–1.2	Moderate	(10, 11)
Bladder	1.1–1.3	Moderate	(1, 11)

Importantly, not all cancers demonstrate increased incidence in patients with diabetes. Prostate cancer, for example, appears to show a neutral or slightly reduced association in several analyses. These variations underscore the need for tumor-specific risk assessment rather than generalized assumptions.

These findings highlight the need for clinical vigilance in selected high-risk populations (5) Similarly, structured hepatocellular carcinoma surveillance should be emphasized in patients with diabetes and advanced NAFLD/NASH (14).

Together, these data support a risk-stratified and multidisciplinary approach integrating metabolic optimization with evidence-based oncologic screening strategies. These observations further reinforce the clinical relevance of risk-stratified screening and metabolic optimization in high-risk populations.

### Biological mechanisms linking type 2 diabetes and cancer

To better understand the clinical implications of this association, it is essential to examine the underlying biological mechanisms linking type 2 diabetes and cancer.

The association between type 2 diabetes mellitus (T2DM) and cancer is supported by several interconnected biological mechanisms that extend beyond epidemiologic correlation. Chronic hyperinsulinemia and insulin resistance are central features of T2DM and may promote tumorigenesis through activation of the insulin receptor and the insulin-like growth factor 1 (IGF-1) axis, with downstream stimulation of PI3K/AKT/mTOR and MAPK signaling pathways that favor cellular proliferation, survival, and anabolic metabolism. These effects are particularly relevant in tissues in which mitogenic and metabolic signaling are tightly coupled (15, 16).

Hyperglycemia may also contribute directly to cancer development and progression. A sustained high-glucose environment can increase oxidative stress, generate advanced glycation end products, alter mitochondrial function, and enhance DNA damage, thereby creating conditions that favor genomic instability and malignant transformation (17, 18). In parallel, excess glucose availability may support cancer cell metabolic plasticity and help sustain glycolytic reprogramming, especially when combined with systemic insulin resistance.

Chronic low-grade inflammation provides another mechanistic bridge between T2DM and cancer. In obesity-associated and insulin-resistant states, adipose tissue, immune cells, and stromal compartments produce pro-inflammatory mediators such as interleukin-6 (IL-6), tumor necrosis factor alpha (TNF-α), and other cytokines that impair insulin signaling while simultaneously promoting angiogenesis, cell survival, and a tumor-supportive microenvironment (18, 19). These inflammatory signals may amplify the transition from metabolic dysfunction to neoplastic progression.

Recent work has further emphasized that the link between diabetes and cancer should be understood in the context of a broader tumor macroenvironment, in which host metabolic fitness, the local tumor microenvironment, and systemic inflammation interact dynamically. Metabolic disorders such as T2DM can shape nutrient availability, immune cell function, adipocyte–tumor crosstalk, and stromal behavior, thereby influencing tumor initiation, progression, metastatic potential, and response to therapy (18).

The bidirectional nature of this relationship is also biologically plausible. Tumors can induce systemic metabolic disturbances through cytokines, extracellular vesicles, altered substrate utilization, and cachexia-related pathways. Pancreatic ductal adenocarcinoma is a particularly important example, as tumor-driven metabolic dysregulation may manifest as new-onset diabetes or rapidly worsening glycemic control before the cancer is clinically recognized (20).

Cancer therapy can further aggravate this metabolic vulnerability. Glucocorticoids worsen insulin resistance and frequently precipitate treatment-related hyperglycemia. In addition, therapies targeting the PI3K pathway may induce hyperglycemia as an on-target effect by disrupting a pathway that is also essential for normal insulin signaling. Immune checkpoint inhibitors, although less commonly, may trigger abrupt insulin-deficient diabetes through immune-mediated beta-cell injury. Together, these treatment effects reinforce the need to interpret cancer-related metabolic dysregulation not as an isolated adverse event but as part of a broader metabolic–oncologic continuum (15, 21–23).

Overall, these mechanisms suggest that the diabetes–cancer association is mediated by a combination of endocrine, inflammatory, redox, immune, and therapy-related pathways. A mechanistic understanding of this interplay supports more integrated clinical strategies, including risk stratification, proactive metabolic monitoring, and multidisciplinary management during cancer treatment (18, 22, 24). These interconnected pathways converge to create a tumor-permissive metabolic and inflammatory environment, reinforcing the biological plausibility of the diabetes–cancer association. Importantly, these mechanistic insights have direct clinical implications.

The growing body of epidemiologic evidence linking type 2 diabetes to increased risk of specific malignancies raises an important clinical question: should diabetes modify current cancer screening strategies? At present, most international cancer screening guidelines do not include diabetes as an independent criterion for intensified surveillance (25, 26). However, the magnitude of association observed for certain cancers—particularly hepatocellular carcinoma and pancreatic cancer—suggests that a risk-stratified approach may be clinically reasonable.

In patients with diabetes and advanced NAFLD or cirrhosis, hepatocellular carcinoma (HCC) surveillance following established hepatology guidelines remains essential (27). Given pooled relative risks exceeding 2.0 for HCC among individuals with diabetes (3, 14), structured surveillance adherence should be emphasized in this subgroup.

New-onset diabetes in individuals over 50 years of age represents a particularly important clinical scenario. Although routine pancreatic cancer screening is not currently recommended for the general diabetic population, the combination of new-onset diabetes with additional red flags—such as unexplained weight loss or rapid deterioration in glycemic control—may warrant individualized evaluation (6).

For colorectal cancer, where the association with diabetes is modest (RR ~1.2–1.3) (7, 28), intensification of screening beyond established age-based recommendations is not currently supported by sufficient evidence. However, ensuring adherence to recommended screening intervals is critical (29).

Overall, while current evidence does not justify universal modification of screening protocols solely based on diabetes status, it supports a personalized, risk-informed approach integrating metabolic profile, organ-specific risk factors, and clinical context.

These insights support a shift from a purely descriptive association toward an integrated metabolic–oncologic model with direct clinical applicability.

### Bidirectional interaction: cancer therapy–induced metabolic deterioration

**Figure 1:**
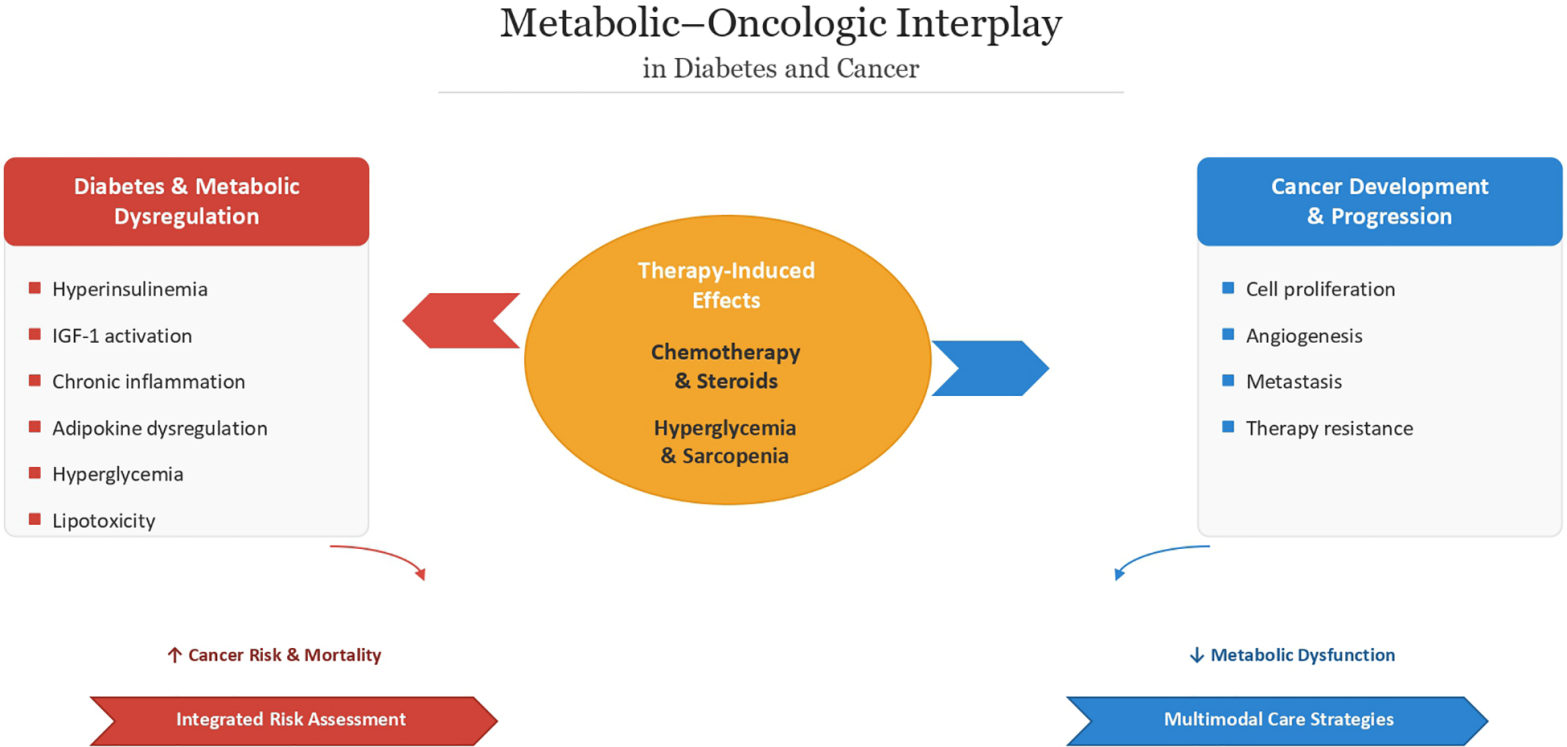
Metabolic–oncologic interplay in diabetes and cancer. Schematic representation of the bidirectional relationship between diabetes-related metabolic dysregulation and cancer development. Hyperinsulinemia, IGF-1 activation, chronic inflammation, adipokine dysregulation, hyperglycemia, and lipotoxicity may contribute to tumor initiation and progression, while cancer therapy–related factors such as chemotherapy, glucocorticoids, hyperglycemia, and sarcopenia may further worsen metabolic dysfunction and clinical outcomes.

The relationship between diabetes and cancer is not unidirectional. While diabetes may increase the risk of certain malignancies, cancer and its treatments can also significantly disrupt metabolic homeostasis, complicating glycemic control and influencing clinical outcomes (**Figure 1**).

Glucocorticoids, widely used as antiemetics and as part of chemotherapy regimens, are a well-recognized cause of treatment-induced hyperglycemia. Steroid-induced insulin resistance may exacerbate preexisting diabetes or precipitate new-onset hyperglycemia, particularly in vulnerable patients (30).

Chemotherapy and systemic cancer progression may further contribute to sarcopenic obesity and metabolic dysregulation. Loss of skeletal muscle mass reduces insulin-mediated glucose disposal, potentially worsening glycemic variability in patients already at metabolic risk (31). In addition, cancer-associated inflammation can amplify insulin resistance through cytokine-mediated pathways (32).

Collectively, these mechanisms underscore the importance of proactive metabolic monitoring during cancer therapy. Integrated collaboration between oncology and endocrinology teams may improve glycemic control, reduce treatment-related complications, and potentially influence oncologic outcomes.

## Practical clinical implications for integrated metabolic–oncologic care

### Integrated metabolic–oncologic care framework

**Figure 2:**
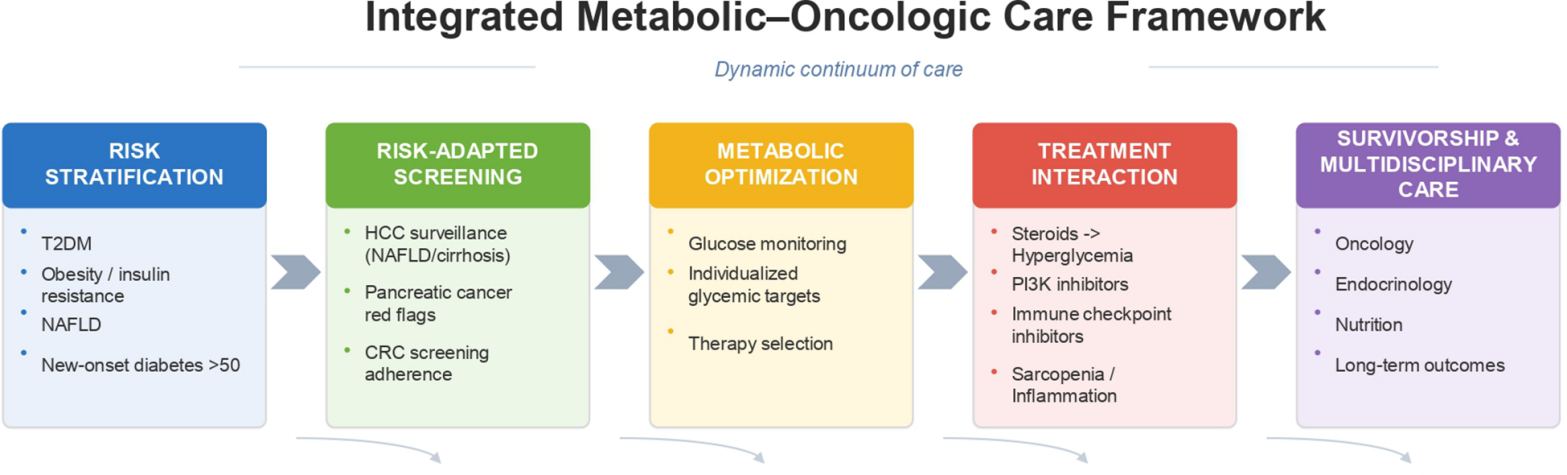
Integrated metabolic–oncologic care framework. Proposed clinical framework linking risk stratification, risk-adapted screening, metabolic optimization, treatment interaction, and survivorship/multidisciplinary care in patients with diabetes and cancer.

The increasing coexistence of type 2 diabetes mellitus (T2DM) and cancer highlights the need for a structured and clinically applicable model that integrates metabolic and oncologic care. Based on current evidence, we propose an integrated metabolic–oncologic care framework that connects risk identification, screening strategies, metabolic optimization, and multidisciplinary management across the cancer continuum (**Figure 2**).

This framework begins with risk stratification, recognizing that patients with T2DM—particularly those with obesity, insulin resistance, or NAFLD—represent a higher-risk population for specific malignancies such as hepatocellular and pancreatic cancer (2, 3, 14, 33). Clinical vigilance is especially warranted in individuals with new-onset diabetes after age 50 accompanied by red flag features, including unexplained weight loss or rapid deterioration in glycemic control (6, 11, 20).

The second component involves risk-adapted screening and early detection. While current guidelines do not universally recommend modified cancer screening based solely on diabetes status (26), selected high-risk patients may benefit from individualized evaluation. Integration of metabolic and oncologic risk factors allows for a more personalized screening approach (6, 18).

The third component focuses on metabolic optimization during cancer therapy. Given the high prevalence of treatment-induced hyperglycemia, proactive glucose monitoring and individualized glycemic targets are essential (11, 21, 33, 34). Selection of antidiabetic therapies should consider both metabolic control and potential oncologic implications, particularly in the context of agents such as glucocorticoids, PI3K inhibitors, and immune checkpoint inhibitors (21–24).

The fourth element is dynamic treatment interaction management, recognizing that cancer therapies may exacerbate metabolic dysfunction, while metabolic status may influence treatment tolerance, toxicity, and potentially oncologic outcomes (15, 16, 18). Continuous reassessment of metabolic parameters during treatment is therefore critical.

Finally, the framework emphasizes multidisciplinary survivorship care, integrating oncology, endocrinology, nutrition, and primary care (28, 34). Long-term management should address both cancer outcomes and cardiometabolic risk, as patients increasingly survive cancer while living with chronic metabolic disease (18, 35).

This integrative model provides a clinically oriented structure to translate mechanistic and epidemiological insights into practical decision-making, supporting a more comprehensive and patient-centered approach to care.

Structured glucose monitoring during cancer therapy is recommended in high-risk settings, and glycemic targets should be individualized according to clinical context (29).

Metformin may offer benefit when not contraindicated (36). Strict adherence to HCC surveillance and multidisciplinary collaboration improve outcomes (27, 37).

The coexistence of diabetes and cancer presents unique challenges that require coordinated management strategies, particularly in several high-risk clinical scenarios. Key clinical situations requiring metabolic monitoring are summarized in **Table 2**. Emerging evidence suggests that proactive metabolic monitoring, individualized antidiabetic therapy selection, and multidisciplinary collaboration can improve clinical outcomes in patients affected by both conditions. Glycemic Monitoring During Cancer Therapy.

**Table 2:** Key clinical situations requiring metabolic monitoring in oncology.

Clinical situation	Metabolic risk	Suggested action
High-dose corticosteroids	Hyperglycemia	Frequent glucose monitoring
PI3K inhibitors	Severe hyperglycemia	Early endocrinology referral
Immune checkpoint inhibitors	Autoimmune diabetes	Monitor glucose and HbA1c
Advanced cancer cachexia or sarcopenia	Insulin resistance and metabolic instability	Nutritional and metabolic assessment

Hyperglycemia is common during cancer treatment due to the metabolic effects of glucocorticoids, chemotherapy, and targeted therapies. Regular glucose monitoring should therefore be implemented in patients receiving treatments known to induce insulin resistance or pancreatic dysfunction (16, 30, 34, 38). Particular attention should be given to patients receiving high-dose corticosteroids, PI3K inhibitors, or immune checkpoint inhibitors, as these therapies may precipitate significant glycemic deterioration or even new-onset diabetes.

### Individualized glycemic targets

Glycemic targets in patients with cancer should be individualized based on life expectancy, treatment intensity, comorbidities, and risk of hypoglycemia (39). For patients receiving intensive oncologic treatment, moderate glycemic control may be preferable to avoid treatment interruptions and minimize infection risk. Conversely, long-term cancer survivors may benefit from tighter metabolic control to reduce cardiovascular and metabolic complications.

### Selection of antidiabetic therapies in cancer patients

Choice of glucose-lowering therapy should consider both metabolic efficacy and potential oncologic implications. Key therapeutic considerations in oncology are summarized in **Table 3**. Metformin has received particular attention due to observational evidence suggesting potential anticancer effects through mechanisms involving AMPK activation, reduced insulin signaling, and inhibition of the mTOR pathway (36, 40). Insulin therapy may be required in patients with severe hyperglycemia or during periods of metabolic stress, although its long−term oncologic implications remain controversial. Newer agents such as GLP−1 receptor agonists and sodium–glucose cotransporter 2 (SGLT2) inhibitors have not demonstrated consistent associations with cancer risk, but long−term oncologic safety data remain limited (43, 44).

**Table 3:** Antidiabetic therapy in the context of cancer care.

Drug class	Oncologic association	Key references
Metformin	Protective observational signal	(36, 40)
Insulin	Neutral/controversial	(41)
Sulfonylureas	Possible increased risk	(42)
GLP-1 RA	No consistent signal	(43)
SGLT2 inhibitors	Limited data	(44)

### Importance of multidisciplinary care

The complex interplay between metabolic dysfunction and cancer therapy underscores the need for integrated care models involving oncologists, endocrinologists, nutrition specialists, and primary care physicians (37, 44). Multidisciplinary management may facilitate early identification of metabolic complications, optimize glycemic control during treatment, and support long−term survivorship care.

## Conclusion

The growing coexistence of diabetes and cancer represents an important clinical challenge with significant implications for patient care. Accumulating epidemiological evidence supports a consistent association between type 2 diabetes and increased risk of several malignancies, particularly hepatocellular and pancreatic cancers. At the same time, cancer and its therapies can substantially disrupt metabolic homeostasis, worsening glycemic control and contributing to treatment-related complications.

These observations reinforce the clinical relevance of the diabetes–cancer relationship and underscore the importance of integrating metabolic considerations into oncologic care. Proactive glucose monitoring, individualized antidiabetic therapy, and careful attention to therapy-induced metabolic disturbances may improve treatment tolerance and clinical outcomes.

As the global burden of both conditions continues to rise, multidisciplinary collaboration between oncology, endocrinology, and nutrition specialists will be essential to optimize management strategies and improve long-term outcomes for patients living with both diabetes and cancer.

Future research should focus on refining risk stratification tools and developing evidence-based strategies to guide integrated metabolic–oncologic care across diverse clinical settings.
